# Primary Hyperparathyroidism: Outcomes of Repeated Imaging After Initial Negative Radiological Localization

**DOI:** 10.7759/cureus.42889

**Published:** 2023-08-03

**Authors:** Dilhara Karunaratne, Nisal Karunaratne, Rishi Vasanthan, Oluwamayowa Ojofeitimi, Emma Owens, Periasamy Sathiskumar, David Till, Paul Kirkland, David Howlett

**Affiliations:** 1 Otolaryngology, Royal Derby Hospital, Derby, GBR; 2 Otolaryngology, Brighton and Sussex Medical School, Brighton, GBR; 3 Otolaryngology - Head and Neck Surgery, Leicester Royal Infirmary, Leicester, GBR; 4 General Internal Medicine, Conquest Hospital, East Sussex Healthcare NHS Trust, St. Leonards-on-Sea, GBR; 5 Radiology, Eastbourne Hospital, East Sussex Healthcare NHS Trust, Eastbourne, GBR; 6 Diabetes and Endocrinology, Conquest Hospital, East Sussex Healthcare NHS Trust, St. Leonards-on-Sea, GBR; 7 Endocrinology, Eastbourne Hospital, East Sussex Healthcare NHS Trust, Eastbourne, GBR; 8 Otolaryngology - Head and Neck Surgery, Eastbourne Hospital, East Sussex Healthcare NHS Trust, Eastbourne, GBR; 9 Interventional Radiology, Eastbourne Hospital, East Sussex Healthcare NHS Trust, Eastbourne, GBR

**Keywords:** preoperative localization, primary hyperparathyroidism (phpt), sestamibi scan, spect-ct, ultrasound

## Abstract

Background: Radiological localization imaging aids in the identification of abnormal parathyroid glands resulting in primary hyperparathyroidism (PHPT), thereby facilitating minimally invasive parathyroid surgery. Sometimes initial imaging may fail to identify the abnormal gland and imaging may therefore be repeated. This study explored patient outcomes of repeated parathyroid localization imaging, after initial negative gland localization, at a United Kingdom institution.

Methodology: Data was retrospectively collected and analyzed for patients with PHPT undergoing repeated imaging during a five-year period (2015-2020). The total number of episodes of scanning, types of scans performed, the time interval between scans and the imaging success of gland localization were recorded. We explored the reasons for repeated imaging and attempted to identify any factors that might predict subsequent positive radiological localization.

Results: A total of 45 patients were identified who underwent repeated localizing imaging after first localizing imaging was negative. Of these, 39 did not undergo surgery despite repeat imaging being undertaken; 11 out of these 39 patients (28%) had subsequent positive localization scans. Again, a large proportion of patients were managed conservatively, despite the repeated sets of imaging being done. Patients undergoing three or four sets of repetitive imaging did not have imaging or surgical success.

Conclusion: A streamlined parathyroid pathway should be followed whereby patients should be triaged for suitability for surgery prior to repeated imaging. A second set of scans should be offered when patients are unsuitable for conservative management and are willing and fit to undergo surgery. There is no merit to repeating imaging more than twice.

## Introduction

Primary hyperparathyroidism (PHPT) is the commonest cause of hypercalcemia and is most often due to a single parathyroid adenoma (80%) [1]. Surgical parathyroidectomy is the recommended treatment for this condition and is indicated if the patient is symptomatic (thirst, frequent or excessive urination or constipation), has end-organ disease (renal stones, fragility fractures or osteoporosis), or has an albumin adjusted calcium level of 2.85 mmol/L or higher [2]. For chosen surgical candidates, the traditional management of PHPT is bilateral neck exploration (BNE), with inspection of all four parathyroid glands and identification and removal of the one(s) that are abnormal [3]. However, in modern practice, improved radiological imaging techniques have allowed for an increased accuracy of localization of a single parathyroid adenoma, allowing the minimally invasive parathyroidectomy (MIP) technique targeted removal of the abnormal parathyroid gland(s) should radiological imaging be positive or concordant (with at least one type of imaging identifying the offending gland). Repeated imaging is discouraged if radiological imaging is discordant (when different imaging modalities show different offending glands) or negative (none of the imaging modalities showing localization). In these cases, patients may be recommended a BNE instead [2].

At our institution East Sussex Healthcare Trust, in 2014, a parathyroid multidisciplinary meeting (MDM) was set up to improve and streamline services for patients [4]. If strict referral criteria are met, patient cases are discussed in order to confirm diagnosis, with access to radiological imaging and coordination of care between experts of different specialties [4]. This helps to come to the most appropriate recommendations regarding further investigations (including further radiological imaging) and whether patient management should be conservative, medical or surgical. This innovative meeting format helps timely and accurate decision making and patient management, with our institution undertaking approximately 40-50 parathyroidectomies a year.

The current study sought to find the outcomes for patients who underwent repeated parathyroid localization imaging, after an initial first set of diagnostic imaging scans that was unsuccessful at localization. We explored the reasons for repeated imaging and attempted to identify any factors that might predict subsequent positive radiological localization. From these findings, the study proposes an optimized management pathway for similar patients. As far as we are aware, this is the first time that the patient outcomes after repeated parathyroid imaging have been explored in detail.

## Materials and methods

From a prospectively recorded database (from records kept at the MDM), details were obtained of adult patients with primary hyperparathyroidism, who had two or more parathyroid localizing sets of scans within a five-year period (2015-2020), after an initial set of negative localization scans, at a single trust (with two district general hospitals, Eastbourne Hospital and Conquest Hospital). Radiological imaging and reports were gathered from the picture archiving and communication system (PACS) and histological reports and biochemical results were gathered from the hospital pathology database. Ethical approval was not required for this study due to its retrospective nature and because it was a service evaluation.

The types of initial localization scans undertaken were a triple combination of ultrasound, technetium-99-labeled sestamibi and single-photon emission computed tomography (SPECT-CT) as is the protocol at our institution. Hyperfunctioning parathyroid glands can be identified on ultrasound as they may be larger in size, hypoechoic, contain fluid or have undergone cystic degeneration [5]. The key parameter indicating the identification of an abnormal gland for a sestamibi scan is increased or sustained uptake on a delayed image as compared to an earlier image [5]. The combination of sestamibi with SPECT-CT can more precisely show the offending gland’s exact anatomical location as compared to planar images [5]. All imaging was performed and reported by specialist head and neck radiologists with expertise in parathyroid imaging, as is the protocol at our institution. Patients were excluded if any components of their records were missing. The total number of episodes of scanning, types of scans performed, the time interval between scans and the imaging success of gland localization were recorded. For gland localization to be defined as negative, two types of imaging needed to be negative. The study explored whether the patients underwent surgery, and if not, the reasons behind the decision.

**Figure 1:**
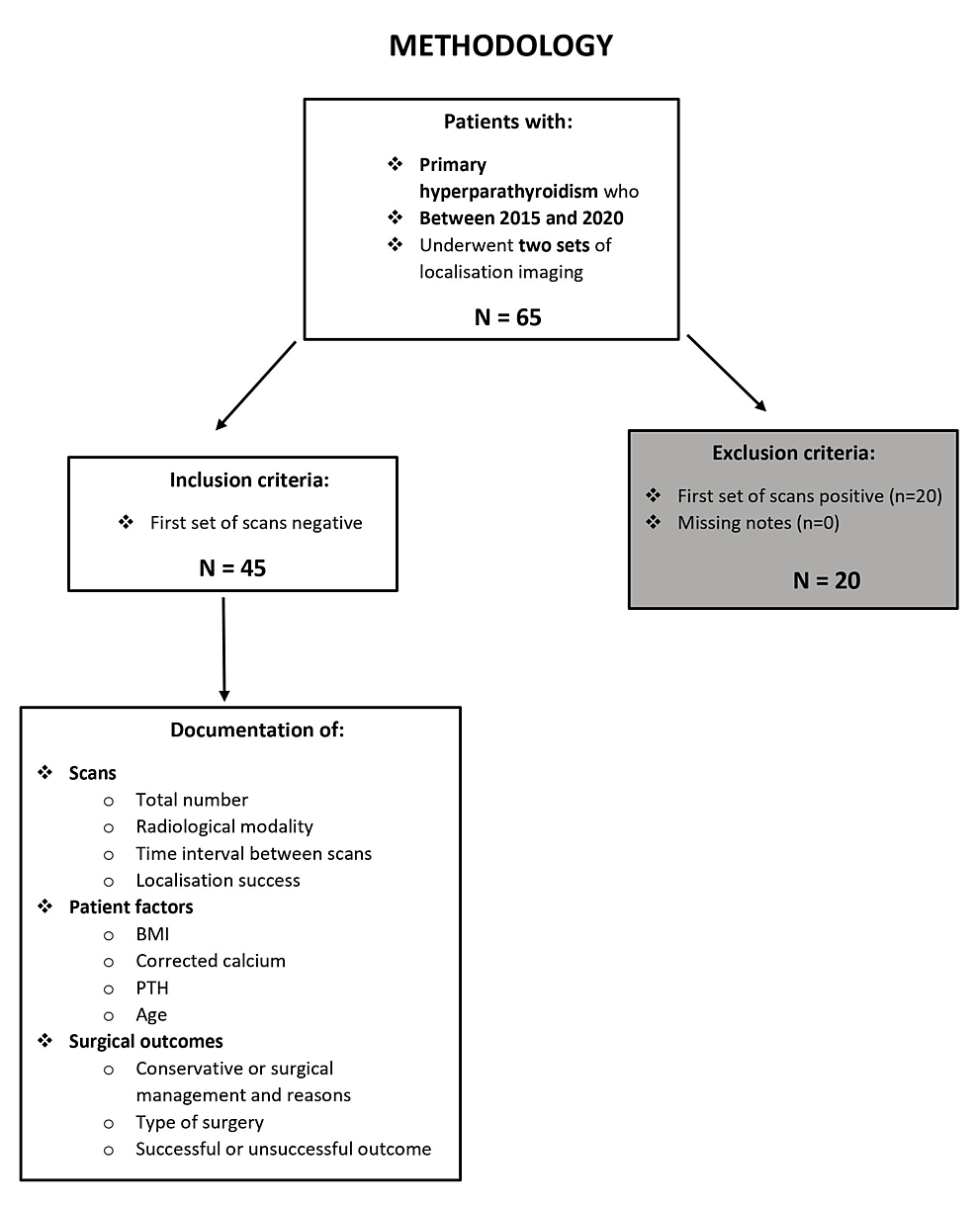
A flow chart showing methodology BMI, body mass index; PTH, parathyroid hormone

Studies suggest that a lower body mass index (BMI) and large gland size correlate with positive gland localization [6,7]. Therefore, patients’ BMI, alongside their corrected serum calcium and parathyroid hormone (PTH) levels (the latter two as surrogate markers for gland size [7,8]), at the time of the imaging episodes, were recorded to see whether these variables might be associated with positive gland localization. Patients’ ages were also compared and time intervals between scans were also measured. This data was analyzed by a medical statistician. The study looked at age, corrected calcium, PTH, BMI, and interval between scans as possible predictors of gland localization success and compared means using a two-sided t-test for independent groups. A flow chart demonstrating the methodology can be seen in Figure 1.

## Results

A total of 45 patients were found to have repeated imaging performed after a negative set of first localizing scans (all patients underwent ultrasound scan, sestamibi and SPECT-CT). All these patients had a biochemical diagnosis of primary hyperparathyroidism and all were deemed to be potential surgical candidates. Out of 45 patients, 38 had an ultrasound scan, sestamibi and SPECT-CT repeated, six had only sestamibi and SPECT-CT and one had SPECT-CT and contrast MRI of the neck. These particular patients’ outcomes form the basis for the results and analysis. There are differences in the type of repeat imaging performed because in some of these patients, sonographic accessibility might have been considered restricted following the initial ultrasound examination and repeating was not thought beneficial. MRI was used in selected patients as a problem-solving tool.

Out of these 45 patients, 38 were female (84.4%) and 7 were male (15.6%). Age ranges of these patients were from 28 to 81; 38 (84.4%) of these 45 patients underwent two sets of localizing scans, 5 (11.1%) had three sets of localizing scans and 2 (4.4%) had four sets of localizing scans.

**Figure 2:**
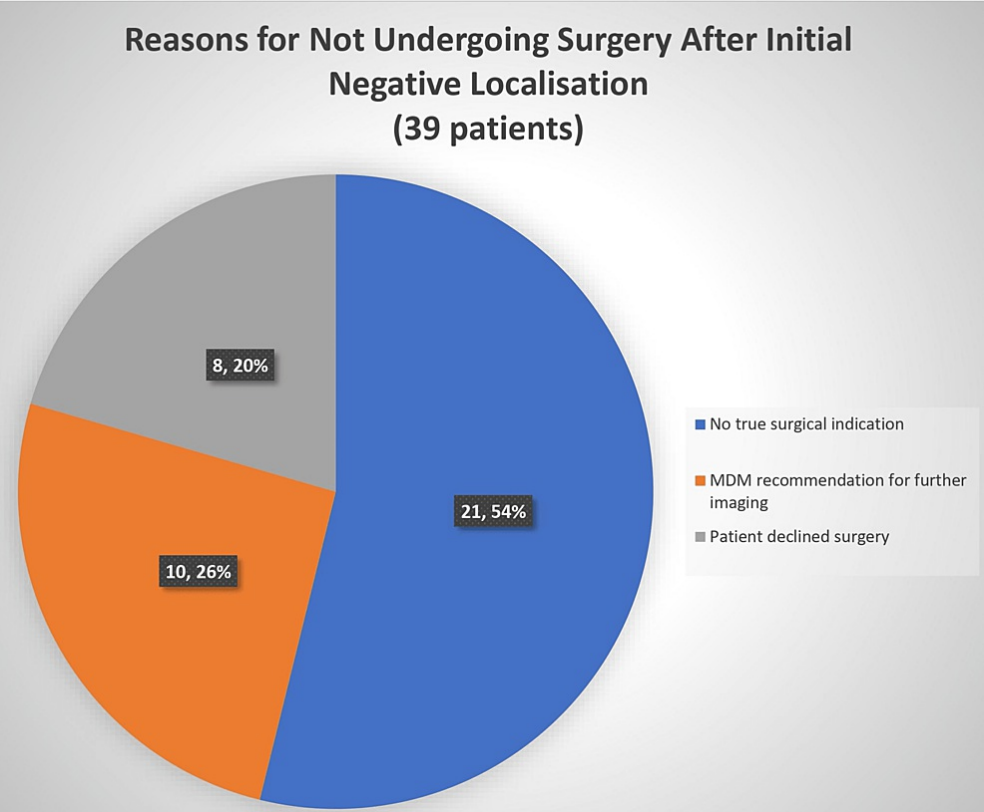
Reasons for why patients who had initial negative localization scans did not undergo surgery MDM, multidisciplinary meeting

Of these 45 patients, 6 (13.3%) underwent unsuccessful BNE surgery after the scans and had another set of localization scans that were negative, and thereafter, four were managed conservatively and two underwent successful repeat BNE. However, 39 (86.7%) of the 45 patients did not undergo surgery for a variety of reasons; 21 (53.8%) did not actually have a clear surgical indication, 8 (20.5%) declined BNE surgery although surgical indications were present and in the remaining 10 (25.6%), in whom surgical indications were present, an MDM decision was taken to repeat imaging to facilitate MIP. This is summarized in Figure 2.

**Figure 3:**
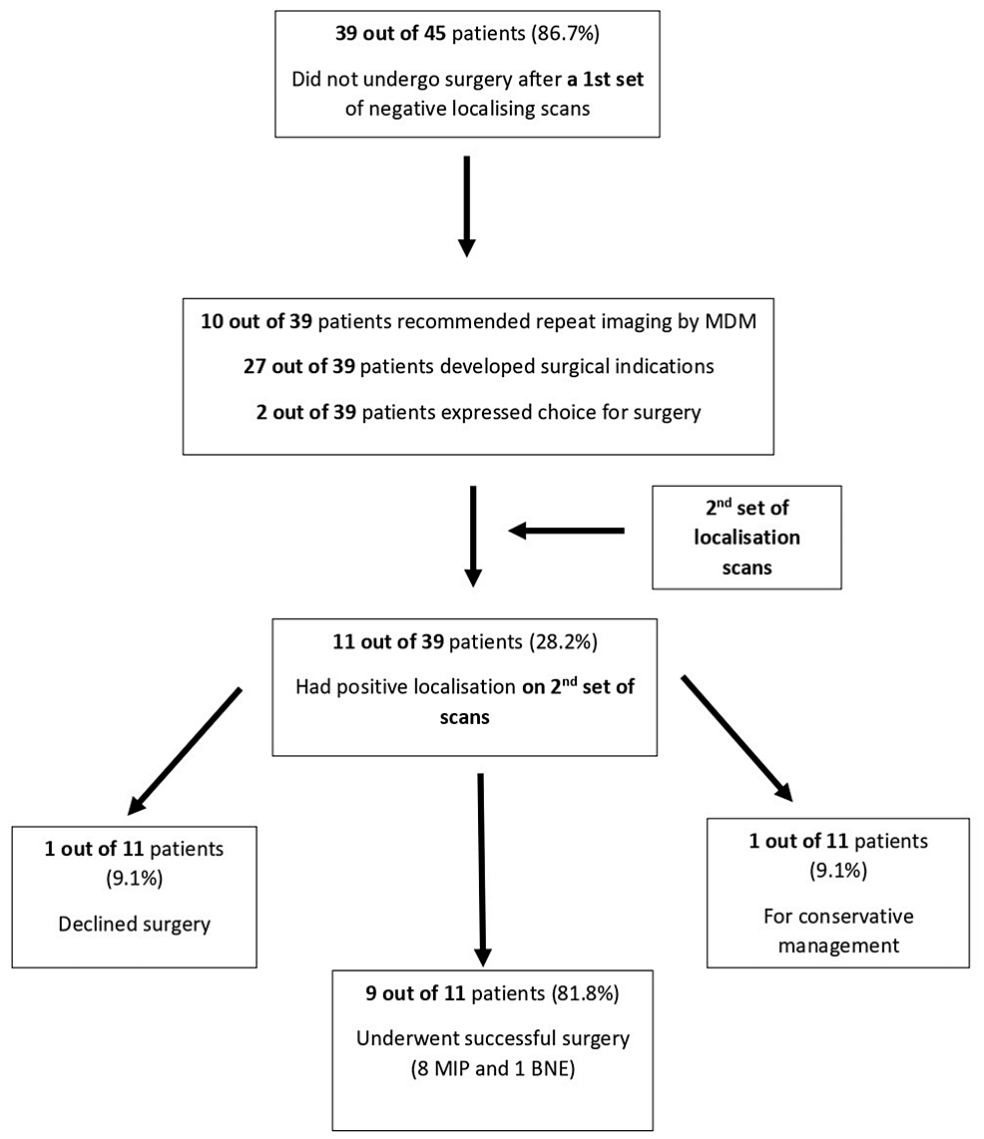
Pathway of patients who had an initial negative localizing scan who did not undergo surgery and had a subsequent positive scan MDM, multidisciplinary meeting; MIP, minimally invasive parathyroidectomy; BNE, bilateral neck exploration

All these 39 patients (who did not undergo surgery) then underwent a second set of localizing scans at various time intervals (10 patients on the recommendation of the MDM, as per Figure 1). A total of 27 out of 39 patients had developed clear surgical indications at the time of repeat imaging; 2 out of 39 patients expressed a personal choice to have surgery over medical management (Figure 3).

Of these 39 patients, 11 (28.2%) had a positive localization on the second set of scans. Of these, 8 (72.7%) patients underwent successful MIP surgery and 1 (9.1%) underwent successful BNE surgery (intraoperative conversion to BNE from MIP). Furthermore, 1 (9.1%) patient declined surgery and 1 (9.1%) patient was managed conservatively as serum calcium levels dropped and remained stable. This is illustrated in Figure 3.

This leaves 28 out of 39 patients (71.8%) who had an initial negative scan, no surgery and subsequently, a second negative scan. Their outcomes are listed in Table 1, which shows there were 17 (60.7%) patients who were decided for conservative management (unfit or declined surgery). Furthermore, 2 (7.1%) patients had been diagnosed with familial hypocalciuric hypercalcemia (FHH) with genetic testing and were managed medically.

**Table 1:** Patient outcomes for those who did not have surgery after a first set of negative scans, but went on to have a second set of scans that were also negative MDM, multidisciplinary meeting; MIP, minimally invasive parathyroidectomy; BNE, bilateral neck exploration; FHH, familial hypocalciuric hypercalcemia

Outcome	Number of patients (%)
Patient declined surgery (for conservative management)	8 (28.6%)
Patient unfit for surgery (for conversative management)	9 (32.1%)
Successful surgery (BNE)	6 (21.4%)
Diagnosed with familial hypocalciuric hypocalcemia (FHH)	2 (7.1%)
Patient was a potential surgical candidate and was advised to have repeat imaging, by the MDM (to facilitate MIP rather than BNE)	3 (10.7%)
Total number of patients	28 (100%)

The patient demographics of the 11 patients who had a first negative localizing scan, no surgery and then a subsequent positive localizing scan were analysed to see whether any patient factor could be predictive of subsequent gland localization success. There was no difference between the groups when comparing the mean average of corrected calcium (p=0.77), BMI (p=0.80), age (p=0.56) and interval between the two scans (p=0.45). However, the 11 patients who had a subsequent positive localizing scan had a higher mean average PTH level (119 pg/mL) compared to the 28 patients with a subsequent negative localizing scan (96.4 pg/mL), but this was not statistically significant (p=0.26), shown in Table 2.

**Table 2:** Comparisons between patients who had two sets of scans and did not undergo surgery BMI, body mass index; PTH, parathyroid hormone

Group	Mean average				
	BMI (kg/m^2^), p=0.8	Age (years), p=0.56	Corrected calcium (mg/dL), p=0.77	PTH (pg/mL), p=0.26	Time interval between scans (months), p=0.45
Negative first set of scans, did not undergo surgery, positive second set of scans (11 patients)	27.9	63	2.8	119.0	18
Negative first set of scans, did not undergo surgery, negative second set of scans (28 patients)	28.4	66	2.8	96.4	16

Five patients underwent three sets of localizing scans; three of these patients had negative localization in all three sets of scans and none underwent surgery, all being managed conservatively (unsuitable for BNE due to medical co-morbidity or declined surgery). The remaining two patients had initial negative scanning and a subsequent positive scan. One of these patients opted for conservative treatment, but later became symptomatic and had another set of up-to-date scans that were negative. The other patient initially declined surgery despite being symptomatic and then later changed their mind and had another set of up-to-date scans that were negative. Both these patients then underwent a fourth set of scans that were negative. Both later underwent successful BNE.

## Discussion

Before the use of minimally invasive techniques, BNE was the reference standard for initial surgery for patients with PTPH [3]. This involves identification of all the parathyroid glands and inspection of common ectopic sites [9], but commits the patient to a less cosmetically favorable incision, higher risk of recurrent laryngeal nerve injury and persistent hypocalcemia [10], alongside the need for inpatient hospital stay, possible requirement of a post-operative drain and failure of the procedure. In modern practice, pre-operative radiological parathyroid localizing techniques have facilitated the use of minimally invasive surgery. This technique is associated with smaller incisions, reduced operative time, quicker patient recovery with reduced post-operative patient complications and comparable outcomes to neck exploration [9]. Therefore, of the two, MIP is the preferred technique.

A variety of imaging modalities are routinely used to localize parathyroid adenomata [11]. Imaging modalities are used in combination, as this is shown to increase the overall sensitivity of gland identification versus when a single imaging modality is used [7,12,13]. The precise combination of imaging modalities for gland localization differs from center to center owing to resource availability and local expertise and is beyond the scope of this paper, but may contribute to different detection rates [14]. At our institution, a triple combination of ultrasound, technetium-99-labeled sestamibi with single-photon emission computed tomography is used in the localization of parathyroid adenomata [4]. As aforementioned, the repeat imaging offered to patients did vary, and this could have affected localization and treatment outcomes.

Multiple factors influence gland localization success. The inferior parathyroid glands tend to have more variable anatomical or ectopic locations [15]. Other causes of failed gland localization may include having a higher or lower number of glands, multiple gland hyperplasia [16] or morphological changes in the thyroid [17]. A specific limitation of ultrasound, often the first-line radiological investigation, is that it is operator dependent and difficult to use in patients with a high BMI, possibly due to increased neck circumference [18]. Localization failure can also occur with parathyroid scintigraphy with early washout of sestamibi from the offending parathyroid gland [9]. Similar factors also apply for the localization failure of SPECT-CT, with differential uptake and retention of the radiotracer within the abnormal parathyroid gland or due to the retention of the tracer within a multinodular thyroid [9].

Some studies show that specific patient characteristics, such as gland size, predict the imaging accuracy of parathyroid localization, with smaller glands correlating with lower success of gland identification [6,7]. It is also demonstrated that the smaller the gland size, the lower the serum calcium level [7,8] and the lower the parathyroid hormone level [8], which may be an important pre-imaging predictor for gland size and hence is a predictor of localization success . Furthermore, a lower BMI significantly correlates with more accurate localization of glands [7].

There is very little literature regarding the success of repeated parathyroid imaging in patients in whom initial scans have failed to localize. However, there are two studies that cite success rates of 41% [19] and 60% [20] on a repeated sestamibi scan following an initial negative scan. The first study included 49 patients and found that factors associated with subsequent positive scans were serum calcium (non-corrected) levels ≥10.5 mg/dL and PTH ≥65 pg/mL, and undertaking the second scan with iodine subtraction [19]. The second study included 40 patients, with no discussion of factors influencing localization success [20]. Although both studies cited higher rates of success compared to the present study and have similar numbers of patients, direct comparisons cannot be made owing to the very different modalities of imaging utilized, between the studies and our practice. However, the initial positive localization rate of the present study (20/45, 44%, see Figure 1) might have impacted subsequent localization success and may partly explain why the repeat positive rate in the present study was lower. These two studies did not explore how a subsequent scan impacted patient management.

**Figure 4:**
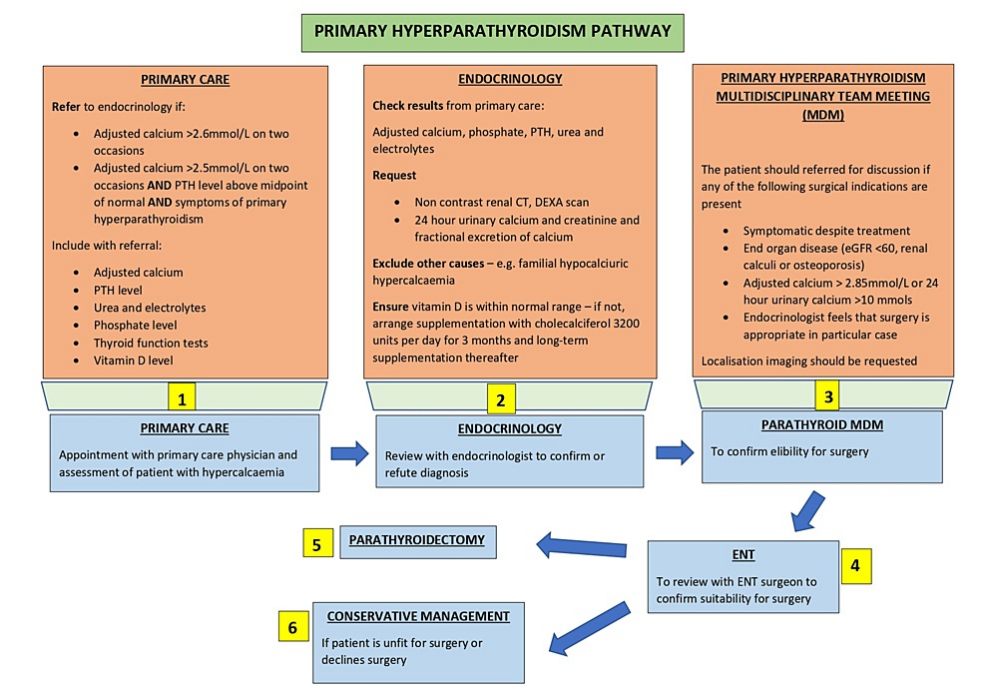
New proposed primary hyperparathyroidism management pathway DEXA, dual x-ray absorptiometry; eGFR, estimated glomerular filtration rate; BMI, body mass index; PTH, parathyroid hormone

Our institution was one of the first in the UK to develop a dedicated, innovative parathyroid MDM, which has proved valuable in the triaging of patients for investigations, discussion of radiological findings, and discussion around the optimum management plan for patients [4]. This includes whether repeat imaging is required and whether a conservative, medical or surgical approach is most prudent. However, the study results show that despite having a dedicated parathyroid MDM, there are some patients who are not true surgical candidates, having imaging repeatedly and then having medical management anyway. A significant proportion of patients also decline surgery. Although reasons for this were not fully documented, it may be that the more invasive BNE option was unacceptable to patients or that the anesthetic and surgical risk posed by major surgery outweighed the benefits, particularly in comorbid patients. Further to this, some of the patients who had undergone repeated imaging were diagnosed with FHH. In these cases, imaging was inappropriate as FHH is a condition managed medically rather than surgically. These results have led to the development of a new primary hyperparathyroidism pathway within our institution, with clear guidance on what medical investigations a patient should undergo and an emphasis on localization imaging being reserved for true surgical candidates only. This new pathway combines the latest National Institute for Health and Care Excellence (NICE) guidelines [2] and an MDM approach, to both optimize the decision-making process and streamline the patient journey (Figure 4) [2,4].

This study demonstrates that some patients (28.2%) with initial negative imaging and no surgery had successful localization on the second set of scans; however, patient factors such as age, BMI, calcium level and time interval between scans had no statistically significant correlation in localization success. Although the mean average PTH levels were (non-significantly) higher in the group with a second set of positive localization scans, the standard deviation was high (47.7 pg/mL). This indicates a high variability of values situated around the true mean and therefore limits the usefulness of triaging imaging based upon PTH levels on an individual basis. Although in some patients, the MDM had recommended repeat imaging after an initial discordant set of scans (to help facilitate MIP over BNE), this data shows that it is difficult to predict which type of patient may have a successful second localization scan and is suitable for repeated imaging. Repeat imaging also needs to be balanced against the radiation exposure risk, estimated to be 5.6 mSv for a sestamibi scan and 12.4 mSv for SPECT-CT [21]. One could tentatively suggest that a second set of localization scans could be considered, but only if patients are true surgical candidates, where FHH has been definitively excluded and patients were both willing and fit enough to undergo surgery. However, there does not appear to be additional benefit to further sets of imaging (three or four) accepting that patient numbers are small in our study.

The strengths of this study include the availability of high-quality imaging and the use of triple imaging modalities undertaken and reported by staff with expertise in head and neck radiology. There are also some limitations present such as the small sample size and patients originating from a small geographical area, which reduces external validity and may prevent generalization of the study findings to the wider population.

## Conclusions

Despite the improvements made to the patient pathway and triaging of patients for appropriate imaging by the dedicated parathyroid MDM, this study has demonstrated that some patients undergo repeat parathyroid imaging without adequate work-up or assessment for suitability for surgery. The new recommended parathyroid pathway aims to streamline the patient journey, by ensuring correct work-up is carried out for primary hyperparathyroidism, reserving parathyroid localization imaging for true surgical candidates only. This pathway is of value to other institutions undertaking parathyroid surgery, by reducing the number of imaging investigations and hence reducing costs and workload within the department and exposure of patients to investigations that do not add value to or change their management.
